# Risk Factors for Peer Victimization among Middle and High School Students

**DOI:** 10.3390/children6010011

**Published:** 2019-01-15

**Authors:** Rebecca A. Vidourek, Keith A. King

**Affiliations:** Health Promotion & Education, University of Cincinnati, Cincinnati, OH 45221-0068, USA; keith.king@uc.edu

**Keywords:** peer victimization, school, school avoidance, peer connectedness

## Abstract

Peer victimization at school is a pressing public health issue. Peer victimization has a deleterious impact on the victim and can lead to lifelong negative outcomes such as depression. The purpose of the present study is to examine peer victimization and potential individual, school, and peer correlates in a national sample of middle and high school students. A secondary data analysis of the School Crime and Safety survey was conducted to investigate study aims. Greater than one in 20 (7.2%) of students reported peer victimization at school. Multiple individual factors were found to increase the odds of victimization including grade level, grades received, and school avoidance among other variables. School and peer factors were also found to be significant. Study findings may be useful to school personnel for reducing peer victimization at school. Specific recommendations for school personnel are offered.

## 1. Introduction

Peer victimization at school is a significant health and educational issue. Peer victimization may include physical, verbal, or psychological harassment or abuse and may contribute to mental and physical health consequences [1,2]. Healthy People 2020 objectives include reducing school-based victimization for adolescents to less than 17.9% of all youth [3]. However, according to the Centers for Disease Control and Prevention (CDC), 1 in 5 youth report experiencing some type of victimization at school [4]. It appears additional research is warranted to understand the factors that contribute to peer victimization. In so doing, the Healthy People 2020 objectives can potentially be met by translating research into initiatives aimed at reducing victimization at school.

Research on victimization suggests more females (23%) than males (19%) report victimization [5]. However, 6% of males report physical victimization, whereas 4% of females report physical bullying. Junior high school students report higher rates of victimization compared to high school students [6]. Students in rural areas also tend to experience less victimization than did students in urban or suburban areas [5]. Additionally, students at highest risk for victimization are perceived as being different, report low self-esteem, are less popular, and do not work well with peers [7].

Concerning school characteristics, previous research found schools with greater than 1000 students have lower rates of victimization than do smaller schools with fewer than 1000 students [8]. Research examining public and private schools found no differences in victimization based on school type. However, higher percentages of students in public schools reported general victimization than did students in private schools [9]. At the classroom level, teachers who attribute victimization to external or outside factors tend to have higher rates of victimization in the classroom [10].

Research also indicates that teacher attitudes and perceptions of bullying play a greater role in classroom victimization than teachers own experiences with victimization. Students who have negative relationships with teachers are more likely to be involved in victimization-related behaviors [11]. In fact, students with high levels of conflict with teachers were found to be rejected at higher levels than did peers with lower levels of conflict with teachers [12]. Conversely, recent research revealed that positive teacher-student relationships resulted in students’ increased bystander behavior by defending victims [13,14].

Peer groups also influence adolescent behavior including peer victimization. Previous research has demonstrated a link between peer behaviors and increases in victimization [15,16]. Specifically, having poor relationships with peers and low levels of support have been identified as risk factors [7,17]. Additionally, participating in negative peer activities such as gang involvement, fighting, and property destruction is identified as increasing victimization related behaviors [18].

On the contrary, having positive relationships with peers is considered a protective factor against victimization [19]. Healthy peer relationships are considered critical for promoting positive mental, emotional and social skills [20] and negative relationships can result in increased aggressive behaviors and reduced school adjustment [21]. Overall, female students tend to have stronger attachments to peers than do male students [22]. Peer relationships also tend to strengthen with age with older students reporting stronger relationships with peers than younger students.

Consequences of peer victimization are numerous and include both short-term and long-term effects. In the short-term, students who experience victimization report being distressed by the bullying incidents [23]. School level consequences of peer victimization also include lower grade point averages and poorer perceived school climate [24]. Peer victimization is also associated with self-harm, depression, suicide, and other mental health disorders [25,26,27]. Adolescents who report peer victimization are also more likely to experience child abuse, injuries from physically fighting, sexual violence, and assault [28,29,30]. Due to the extensive negative consequences associated with peer victimization, additional research is warranted to determine factors associated with victimization.

### Purpose of the Present Study

The present study is designed in part based on the risk and protective factor model. Although the model was originally applied to alcohol and drug use [31], it is now applied to multiple youth health behaviors including peer victimization [32,33]. With the extensive nature of peer victimization as well as the significant negative consequences associated with such behavior, additional research is needed on factors contributing to peer victimization at school. Utilizing the risk and protective factor model as a guide, identifying factors can lead to early targeting of predictors of victimization [34]. In addition, this study is also guided by the social-ecological systems theory [35]. Originally developed by Bronfenbrenner, this theory posits that child development occurs within multiple social systems that influence the child and overall child growth. In this study, the social systems examined include school, teacher, and peer systems and the influence on peer victimization.

The following study examines peer victimization using a nationally representative sample of participants, which is a strength of the study. In addition, few studies have examined potential links between peer victimization and peer connectedness and gangs at school. Thus, the present study investigated associations between individual factors, school characteristics, and peer factors and peer victimization. The present study tested the following hypotheses:

**Hypothesis** **1:** *Males will be significantly more likely than females to report being victimized by peers.*

**Hypothesis** **2:** *High school students will be significantly more likely than junior high school students to report being victimized by peers.*

**Hypothesis** **3:** *Students who receive lower grades (Cs/Ds/Fs) will be significantly more likely than students who report higher grades (As/Bs) to report being victimized by peers*.

**Hypothesis** **4:** *Students who frequently engage in negative school behaviors (skipping class, avoiding school/school activities, and experiencing distractions in class) will be significantly more likely than students who do not frequently engage in negative school behaviors to report being victimized by peers.*

**Hypothesis** **5:** *Students who attend public schools will be significantly more likely than students who attend private schools to report being victimized by peers.*

**Hypothesis** **6:** *Students who attend schools large in enrollment size will be significantly more likely than students who attend schools small in enrollment size to report being victimized by peers.*

**Hypothesis** **7:** *Students who attend schools with a high percentage of students receiving free/reduced lunch will be significantly more likely than students who attend schools with a low percentage of students receiving free/reduced lunch to report being victimized by peers.*

**Hypothesis** **8:** *Students who are involved in gangs will be significantly more likely than students who are not involved in gangs to report being victimized by peers.*

**Hypothesis** **9:** *Students who have low peer connectedness will be significantly more likely than students who have high peer connectedness to report being victimized by peers*.

In addition, the following research questions were examined: (1) What is the extent of peer victimization in a national sample of students 12 to 18 years old?; (2) Does peer victimization differ based on individual factors including sex, grade level, grades received, skipping class, avoiding school/school activities, and experiencing distractions in class?; (3) Does peer victimization differ based on school characteristics including school type, enrollment size, and percentage of students receiving free/reduced lunch?; and (4) Does peer victimization differ based on peer factors including gangs in school and peer connectedness?

## 2. Materials and Methods

### 2.1. Participants

Participants in this study were 12 to 18 years old and currently enrolled in primary, middle, and high schools. All participants were enrolled in school in the previous six months before the questionnaire was administered. All participants were students enrolled in a school that leads to a high school diploma. Participants were not eligible if they dropped out of school, were suspended or expelled from school, or who were absent from school for an extended period of time in the previous six months before the survey. Further, home school students were also excluded due to the nature of the survey.

### 2.2. Instrumentation

This study is a secondary data analysis of the 2015 National Crime Victimization Survey (NCVS): School Crime Supplement (SCS) for students 12 to 18 years of age. The SCS is a supplemental survey linked to the NCVS survey response, which examines the nature and extent of crime nationwide. Participants are recruited from all 50 states to complete the questionnaire. The SCS assesses school-related victimization in order to inform professionals involved in policy, research and others working with crime and safety at school.

The SCS includes 8 sections. For the purpose of this study, the following sections were used in the present study: (1) Peer Victimization; (2) Individual/Student Factors; (3) School Characteristics; and (4) Peer Factors. Peer victimization was assessed by 2 items and requested students to report if they had been threatened with harm by another student at school and pushed, shoved, tripped, or spit on by another student at school. Students who were victimized were operationally defined as being threatened with harm at school and pushed, shoved tripped, or spit on at school. The victimization variable was dichotomized into two categories: 0 = not victimized and 1 = victimized. Individuals’ factors were assessed by five items including grade level, grades received, school avoidance, classroom distraction, and skipped class past 30 days. School characteristics were assessed by three items including school type (private/public), enrollment size, and percentage of students on free/reduced lunch. Peer factors were examined using two items: Presence of gangs in school and peer connectedness. Peer Connectedness was assessed by three items assessing if students at school cared about them, listened to them, and believed they will be successful. A composite peer connectedness score was developed and dichotomized into two categories: Yes, connected/no, disconnected.

### 2.3. Procedures

The SCS Questionnaire was developed by the Bureau of Justice Statistics, the National Center for Education Statistics, and the U.S. Census Bureau. Participants were selected by the US Census Bureau by randomly selecting households and dividing them into rotations or groups. All age-eligible individuals became part of the panel. Over a three year period, study participants were interviewed once every six months for a total of seven interviews. The first interview is held face to face whereas the remaining SCS are administered to eligible participants using computer-assisted telephone interviewing methods. Participants are informed that all responses are confidential.

### 2.4. Data Analysis

All data analysis was computed with SPSS Version 24.0. Demographic characteristics were computed via frequency distributions, means, and standard deviations. A series of chi-square analyses and odds ratios were computed to determine differences in peer victimization based on individual factors, school characteristics, and peer connectedness. All significant univariate items were subsequently retained and included in a final logistic regression model. An alpha level of 0.01 was set a priori to determine significant differences.

## 3. Results

### 3.1. Demographic and School Characteristics

Among the households that participated in the NCVS, there were 9372 individuals ages 12–18 who were eligible to complete the SCS in 2015. Among those youth ages 12–18 who were found eligible for the 2015 SCS, a total of 4767 completed the survey. Regarding sex, 41.4% were male and 58.6% were female. Regarding grade level, 42.1% were in middle school and 57.9% were in high school. Concerning race, 80.3% were white and 19.7% were nonwhite. One in four (23.4%) reported being of Hispanic origin. The overwhelming majority of students attended public schools (93.2%) and 6.8% attended private. Concerning school characteristics, 45.4% of students attended a school with more than 1000 students whereas 54.6% of students attended a school with less than 1000 students.

### 3.2. Peer Victimization

In this sample, a total of 7.2% of participants reported being threatened with harm, pushed, shoved, tripped or spit on at school.

### 3.3. Individual Factors and Peer Victimization

With the exception of sex, all individual factors examined in the analysis were found to be significant. Results indicated that peer victimization did not significantly differ based on sex, (OR = 1.091 (95% CI = 0.791, 1.506), *p* = 0.595). Results indicated the following individual factors increased the odds for peer victimization: Being in middle school, receiving Cs/Ds/Fs, recently (past 30 days) skipping class, avoiding school/school activities, and being distracted in class (Table 1). Odds ratios of note included students who were distracted in school were 4.972 times more likely to experience peer victimization and students who school/school activities were 14.442 times more likely to report peer victimization.

### 3.4. School Characteristics and Peer Victimization

Results indicated that students attending a school with less than 1000 students were at increased odds for peer victimization (Table 2). In fact, students attending a school with less than 1000 students were 1.693 times more likely to experience peer victimization compared to students attending a school with more than 1000 students. School type (public vs. private) and percentage of students receiving free or reduce lunch (50% or more vs. 50% of less) was not significantly correlated to peer victimization.

### 3.5. Peer Factors and Peer Victimization

Results demonstrated that gangs in school was significantly associated with peer victimization (Table 3). In fact, students reporting gangs in school were 3.822 times more likely to report peer victimization. Concerning peer connectedness, students reporting that they were not connected to peers were 2.582 times more likely to report peer victimization.

### 3.6. Final Logistic Regression Model for Peer Victimization

The final logistic regression model significantly predicted peer victimization (omnibus chi-square = 136,227, df = 8 *p* < 0.001) and accounted for 7.4% (Cox and Snell R^2^) to 18.8% (Nagelkerke R^2^) of the variance in peer victimization (Table 4). Significant predictors included grade level, grades received, distracted in class, enrollment, and gangs in school.

## 4. Discussion

The present study found greater than one in 20 students reported peer victimization. Specifically, students reported being threatened with harm, pushed, shoved, tripped or spit on by students. A previous study found victimization to be 19% in a national samples of middle and high school students whereas a meta-analysis of bullying found a mean rate of 35% [4,36]. As victimization was delimited to threats and pushing, shoving, tripping or spiting, this may explain why victimization was lower in this study. This finding is still concerning as the consequences of victimization are numerous and no student should be victimized at school.

With the exception of sex, all individual factors were found to be significantly associated with victimization in the univariate analysis and all but skipping class were significant in the final model. Based on the findings, Hypothesis 1: Males will be significantly more likely than females to report being victimized by peers was rejected. However, the following hypotheses were accepted: Hypothesis 1: Middle school students will be significantly more likely than high school students to report being victimized by peers; Hypothesis 2: Students who receive lower grades (Cs/Ds/Fs) will be significantly more likely than students who report higher grades (As/Bs) to report being victimized by peers; Hypothesis 3: Students who frequently engage in negative school behaviors (skipping class, avoiding school/school activities, and experiencing distractions in class) will be significantly more likely than students who do not frequently engage in negative school behaviors to report being victimized by peers. Long-term approaches recommended by the Centers for Disease Control and Prevention may address some of the individual level factors. For example, recommendations include improving youth communication skills, critical thinking and problem-solving skills and social-emotional skills [37]. Schools can incorporate these skills into classroom lessons, school activities, and targeted programming. For students who are distracted in class, avoid school and school/activities and skip class, these strategies may provide them with skills necessary to deal with victimization and increase their ability to reach out for help if they are victimized. It may also provide the tools needed for students to intervene when they see others being victimized at schools.

Concerning school characteristics, no significant differences in public and private schools and percentage of students on free/reduced lunch were found. Therefore, the following hypotheses were rejected: Hypothesis 4: Students who attend public schools will be significantly more likely than students who attend private schools to report being victimized by peers; and Hypothesis 6: Students who schools with a high percentage of students receiving free/reduced lunch will be significantly more likely than students who attend schools with a low percentage of students receiving free/reduced lunch to report being victimized by peers.

The present study found enrollment size was significant in both the univariate analysis and in the final model. Students enrolled in schools with fewer than 1000 students were 1.6 times more likely to experience peer victimization compared to students enrolled in schools with greater than 1000 students. Therefore, Hypothesis 5 was accepted: Students who attend schools large in enrollment size will be significantly more likely than students who attend schools small in enrollment size to report being victimized by peers. For school personnel, the CDC recommends multiple approaches to preventing and reducing peer victimization. One such approach involves school-wide programs targeting school climate, improving adult supervision of students and clear and firm rules against bullying [38]. It is possible smaller schools lack the necessary resources to carry out such initiatives. Assessing school strengths and weaknesses may be one strategy to identify gaps in victimization prevention. Positive school climate is associated with reduced victimization and promotes school connectedness [39]. Moreover, adult supervision, particularly in hallways, bathrooms, and the cafeteria, is crucial to preventing victimization. Schools should create plans for supervision throughout the school and educate all school staff on the importance of supervision in reducing victimization. As part of any victimization plan, schools also need clear, firm, and enforced rules against victimization. Ensuring students, teachers, and staff are informed of such rules is also critical.

Specific peer variables played a role in student experiences of victimization. Students who reported gangs were in the school were 3.8 times more likely to experience victimization compared to students who did not have gangs in the school. In the final model, students with gangs in school were 3 times more likely to experience peer victimization than did their counterparts. Therefore, Hypothesis 7 was accepted: Students who are involved in gangs will be significantly more likely than students who are not involved in gangs to report being victimized by peers. It may be that gangs at school contribute to an overall increase in all types of violence and victimization in the school setting. It may also be that students in schools with gangs do not feel positively connected and seek out methods of connecting with others through gang membership. In addition, students who did not feel connected to peers were 2.5 times more likely to experience victimization compared to peers who felt connected. However, peer connectedness was not significant in the final model. Therefore, Hypothesis 8, students who have low peer connectedness will be significantly more likely than students who have high peer connectedness to report being victimized by peers, was not rejected in the univariate analysis yet rejected in the final model. Feelings of disconnectedness result in a variety of negative outcomes for youth. One recent study found disconnected male students were more likely to be aggressive and use drugs and disconnected female students were more likely to suffer from depression [39].

Building positive peer relationships may be one critical strategy to reducing peer victimization. It also may be a point of intervention by identifying peers who feel disconnected from classmates and working to positively involve disconnected students in school activities. Working with disconnected students to recognize activities they may enjoy and including them in such activities may reduce victimization by connecting them to peers. It may also prevent negative outcomes such as gang involvement while also promoting a positive connection to schools and peers.

### Limitations

The following study limitations should be noted. Participants in this study were delimited to students from 5th–12th grade. Thus, caution should be exercised when generalizing these findings to other grades. All study data is self-reported; thus, some students may have responded in a socially desirable way. In addition, the questionnaire is cross-sectional in nature; therefore, causal relationships cannot be determined. Further, the SCS provides key definitions on the survey, which some research suggests may lead to an underreporting of some behaviors. Also, the Bureau of Justice Statistics uses coefficients of variance to determine reliability. Therefore, future researchers may wish to conduct additional testing of reliability including a confirmatory factor analysis.

## 5. Conclusions

The present study found greater than 1 in 20 students reported peer victimization. Multiple factors were found to be associated with victimization at the individual, school, and peer level. Specifically, students in middle school, receiving Cs/Ds/Fs, being distracted in class, lower school enrollment, and having gangs in school all increased the odds for victimization. Based on study results, there are several practical implications. Middle school may be an ideal time to implement victimization prevention and intervention programs. Targeting this group may reduce victimization and prevent victimization from continuing into the high school years. Another at risk group includes students with lower grades. It is possible programming and other initiatives are targeted to high achieving students. Formulating special efforts to include lower achieving students in classroom and school activities may reduce victimization while also improving staff and peer relationships. Similarly, efforts are also warranted for students experiencing class distraction. It may be that the victimization is actually contributing to the class distraction. Additional research is needed to determine potential relationships. For school enrollment, schools with fewer than 1000 students may consider implementing the CDC strategies to reduce victimization at school. Specific guidelines for decreasing victimization are available and, if necessary, smaller schools can collaborate with community agencies and groups to establish and implement plans. In addition, not surprisingly, gangs at school increased victimization. All schools should assess the presence of gangs in school and take appropriate steps to reduce gang membership. Similarly, to school size, these schools may need to partner with local law enforcement or community groups to reduce gang affiliation among students.

In addition to the above strategies, global approaches that address individual, school, and peer factors are warranted to prevent and reduce peer victimization at school. Identifying students who are at risk based on individual factors may allow for intervention. Long-term, school-based approaches may involve creating and promoting firm rules against victimization, improving staff supervision of students and promoting social and emotional skills for students. Targeting victimization at multiple levels is also important as individual, school, and peer factors all contributed to victimization. Future research may examine additional peer and school level factors as a means of further understanding school-based victimization among youth.

## Figures and Tables

**Table 1:** Individual Factors and Peer Victimization.

Item	Peer Victimization		OR	CI	*p*
	No *n* (%)	Yes *n* (%)			
**Grade Level**					
High School ^a^	1265 (95.2)	64 (4.8)	2.291	(1.654, 3.175)	<0.001
Middle School	854 (89.6)	99 (10.4)			
**Grades Received**					
As/Bs ^a^	1803 (94.1)	113 (5.9)	2.695	(1.890, 3.843)	<0.001
Cs/Ds/Fs	296 (85.5)	50 (14.5)			
**Skipped Class Past Month**					
No ^a^	2010 (93.4))	143 (6.6)	2.362	(1.428, 3.907)	0.001
Yes	119 (85.6)	20 (14.4)			
**Avoided School/School Activities**					
No ^a^	2123 (93.5)	147 (6.5)	14.442	(7.358, 28.347)	<0.001
Yes	18 (50.0)	18 (50.00)			
**Distracted in Class**					
No ^a^	1182 (97.3)	33 (2.7)	4.972	(3.363, 7.350)	<0.001
Yes	951 (87.8)	132 (12.2)			

^a^ Indicates Referent.

**Table 2:** School Characteristics and Peer Victimization.

Item	Peer Victimization		OR	CI	*p*
	No *n* (%)	Yes *n* (%)			
**School Type**					
Private ^a^	144 (95.4)	7 (4.6)	1.629	(0.750, 3.539)	0.213
Public	1995 (92.7)	158 (7.3)			
**Enrollment Size**					
More than 1000 students ^a^	936 (94.7)	52 (5.3)	1.693	(1.201, 2.386)	0.002
Less than 1000 students	1115 (91.3)	106 (8.7)			
**Percentage of Students on Free/Reduced Lunch**					
Less than 50% ^a^	1023 (92.9)	78 (7.1)	1.125	(0.810, 1.562)	0.482
More than 50%	886 (91.2)	76 (7.9)			

^a^ Indicates Referent.

**Table 3:** Peer Factors and Peer Victimization.

Item	Peer Victimization		OR	CI	*p*
	No *n* (%)	Yes *n* (%)			
**Gangs in School**					
No ^a^	1591 (94.5)	93 (5.5)	3.822	(2.576, 5.670)	<0.001
Yes	188 (81.7)	42 (18.3)			
**Peer Connectedness**					
Yes ^a^	1994 (93.4)	141 (6.6)	2.582	(1.573, 4.239)	<0.001
No	115 (84.6)	21 (15.4)			

^a^ Indicates Referent.

**Table 4:** Final Logistic Regression Model for Peer Victimization.

Item	β	S.E.	Wald	Exp *b*	CI
**Grade Level**	**0.792**	**0.228**	**12,084**	**2.208**	**(1.413, 3.451)**
**Avoidance**	**1.623**	**0.453**	**12,858**	**5.071**	**(2.088, 12,315)**
**Grades Received**	**0.694**	**0.230**	**9.120**	**2.002**	**(1.276, 3.141)**
Recently Skipped Class	0.625	0.336	3.466	1.869	(0.968, 3.611)
**Distracted in Class**	**1.197**	**0.243**	**24,211**	**3.310**	**(2.055, 5.322)**
**Enrollment**	**0.482**	**0.238**	**4.094**	**1.619**	**(1.015, 2.583)**
**Gangs in School**	**1.109**	**0.242**	**20,930**	**3.032**	**(1.885, 4.877)**
Peer Connectedness	0.445	0.333	1.787	1.560	(0.813, 2.994)

Significant variables bolded. The model significantly predicted peer victimization (omnibus chi-square = 136.227, df = 8 *p* < 0.001) and accounted for 7.4% to 18.8% of the variance in peer victimization.
